# Compassionate mesenchymal stem cell treatment in a severe COVID-19 patient: a case report

**DOI:** 10.3325/cmj.2021.62.288

**Published:** 2021-06

**Authors:** Dragan Primorac, Sanda Stojanović, Stipić, Marko Strbad, Lenart Girandon, Ariana Barlič, Mileva Frankić, Ivo Ivić, Daniela Marasović, Krstulović, Irena Jukić, Beata Halassy, Ana Hećimović, Vid Matišić, Vilim Molnar

**Affiliations:** 1 St. Catherine Specialty Hospital, Zagreb/Zabok, Croatia; 2 Eberly College of Science, The Pennsylvania State University, University Park, State College, PA, USA; 3 The Henry C. Lee College of Criminal Justice and Forensic Sciences, University of New Haven, West Haven, CT, USA; 4 Medical School, University of Split, Split, Croatia; 5 School of Medicine, Faculty of Dental Medicine and Health, Josip Juraj Strossmayer University Osijek, Osijek, Croatia; 6 School of Medicine, Josip Juraj Strossmayer University Osijek, Osijek, Croatia; 7 Faculty of Medicine^,^ University of Rijeka, Rijeka, Croatia; 8 Medical School REGIOMED, Coburg, Germany; 9 Department of Anesthesiology and Intensive Care, Clinical Hospital Split, Split, Croatia; 10 Educell Ltd., Trzin, Slovenia; 11 Biobanka Ltd, Trzin, Slovenia; 12 Department of Infectious Diseases, University Hospital Split, Split, Croatia; 13 Department of Internal Medicine, University Hospital Split, Split, Croatia; 14 Croatian Institute of Transfusion Medicine, Zagreb, Croatia; 15 Center for Research and Knowledge Transfer in Biotechnology, University of Zagreb, Zagreb, Croatia; 16 Center of Excellence for Virus Immunology and Vaccines, CERVirVac, Zagreb, Croatia

## Abstract

COVID-19 presentations range from cold-like symptoms to severe symptoms with the development of acute respiratory distress syndrome (ARDS). We report on a severe COVID-19 patient who was mechanically ventilated and who developed ARDS and bacterial infection. Because of rapid clinical deterioration and the exhaustion of other treatment options, the family and attending physicians requested a compassionate use of adult allogeneic bone marrow-derived mesenchymal stem cells (MSC) in addition to commonly used immunosuppressive, antiviral, and supportive therapy. The clinical course is discussed thoroughly, with a special emphasis on the safety and effect of MSC therapy. Compassionate MSC treatment, given in three rounds, affected ARDS regression. The patient was discharged from the intensive care unit after 31 days and from hospital after 49 days in a good general condition. MSC treatment was not associated with any side effects and was well tolerated in a three-week period; therefore, it should be studied in larger trials and considered for compassionate use.

We report on a case of severe COVID-19 in a 50-year-old male patient who was treated with an infusion of parenteral mesenchymal stem cells (MSC) (Table 1). The pathophysiologic mechanism of COVID-19 is not yet fully understood, but some patients experience a strong immune response, with a release of pro-inflammatory cytokines and chemokines causing a “cytokine storm.” These patients present with a severe clinical course, acute respiratory distress syndrome (ARDS), and require mechanical respiratory support (1,2). Studies investigating MSC in COVID-19 treatment have shown good results in reducing the serum levels of pro-inflammatory cytokines and chemokines, which are the main driver of ARDS in these patients (3).

**Table 1:** Timeline of the disease and treatment course

Day of hospitalization	Clinical, radiological, and laboratory findings
1st day of the disease	First presented to the emergency department of a regional COVID-19 center after five days of home isolation and COVID-19 treatment. Symptoms: fever of up to 38.5 °C, myalgia, and diarrhea. Clinical findings: eupneic, blood oxygen saturation (Sao_2_) 97%. Laboratory results: elevated C-reactive protein (CRP) 11.3 mg/L. Chest x-ray: bilateral basal and perihilar opacifications. Treatment: released for home treatment with 32 mg methylprednisolone for 7 d in a de-escalating pattern.
1st (4th day of the disease)	Presented with worsening symptoms. Methylprednisolone dose escalated to 64 mg and added amoxicillin and clavulanic acid after finishing azithromycin. Symptoms: fever over 39 °C, dry cough Clinical findings: Sao_2_ 94%. Laboratory results: elevated leukocytes (10.9 × 10^9^/L) with neutrophil predominance (90.4%, lymphocytes 5.7%), mildly elevated CRP 24.9. Chest computed tomography (CT): extensive bilateral ground-glass opacities. Treatment: admission to a specialized COVID-19 ward, remdesivir (200 mg iv), methylprednisolone 240 mg, enoxaparin 0.6 mL, moxifloxacin 400 mg, and supportive antipyretic therapy.
2nd	Oxygen supplementation was started with 2 L/min nasal cannula.
3rd	The condition continued to worsen (Sao_2_ 91%). High-flow oxygen therapy (35 L/min) initiated.
6th	Clinical deterioration with dyspnea. Sao_2_ 87% Laboratory findings suggested an increased inflammatory response and severe thrombocytopenia (10 × 10^9^/L). CT pulmonary angiography showed no signs of pulmonary embolism but showed a progression of bilateral consolidations. Treatment: high-flow oxygen increased to 45 L/min. Hematologist consulted: immunogenic thrombocytopenia. Intravenous immunoglobulins (400 mg/kg), methylprednisolone changed to 100 mg.
7th	Referral to a COVID-19 intensive care unit (ICU). Mechanical ventilation started (synchronized intermittent mandatory ventilation f/24min, TV 440 mL; first FiO_2_ 100%, then gradually decreased to 80%). FiO_2_ was gradually reduced to 50% the same day.
8th	The patient was given a dose of convalescent plasma. Immeasurably high levels of D-dimers (>35.2 mg/L).
9th	The hospital ethics and drug committees approved mesenchymal stem cells (MSC) compassionate treatment. The first dose of allogenic bone marrow-derived MSC (10^6^ cells/kg) was given.
12th	The second dose of allogenic bone marrow-derived MSC was given. The patient was transferred to a tertiary hospital center for further treatment. The therapy was continued, and the patient was mechanically ventilated.
13th	Continued to clinically worsen. Sao_2_ below 90%. Later the same day developed subcutaneous emphysema. Laboratory results: reduction of inflammatory parameters. Bronchoscopy: abundant yellowish secretion, sent for microbiologic evaluation. Chest CT: extensive airspace opacifications and pneumomediastinum. Low ventilation compliance (tidal volumes below 350 mL) with decreasing Sao_2_ levels indicating acute respiratory distress syndrome. Microbiologic culture of the bronchial aspirate: methicillin-resistant *Staphylococcus aureus* and *Acinetobacter Baumannii*. Treatment: recruitment maneuver, high positive end-expiratory pressure (PEEP) (Hamilton-Intellivent ASV) with low driving pressure (below 14 cm H_2_O). PEEP was continuously reduced in the following days. Antibiotic treatment (colistin 9 million i.u. initially, the next and following days 2 × 4.5 million i.u., fosfomycin 3 × 8 g, and linezolid 2 × 600 mg)
15th	Repeated bronchoscopy showed the same findings. Chest x-ray: no reduction in subcutaneous emphysema.
16th	The third dose of allogenic bone marrow-derived MSC was given. Chest x-ray: regression of opacifications. Later the same day paroxysmal hypertension >200 mm Hg and tachycardia >140/min. Echocardiography: analysis impeded by pneumomediastinum, normal left ventricle morphology, ejection fraction of 50%.
17th	The patient was extubated and put on high-flow oxygen (60 L/min).
19th	Clinical improvement. Oxygenation changed from high-flow oxygen to nasal cannula. Verticalized in the course of physical therapy.
20th	Lower urinary tract pain. Laboratory findings: bacteriuria. Blood cultures: positive for gram-negative bacilli. Treatment: ceftazidime/avibactam (3 × 2.5 g iv).
22nd	Clinically stable. Leukocytes returned to physiological levels.
23rd	Chest CT: regressive dynamic of airspace opacifications.
30th	Able to perform basic physical activities on his own. No oxygen supplementation needed. CRP levels normal. All antibiotics discontinued.
31st	Transferred to a clinic for physical medicine for further rehabilitation.
49th	Discharged from hospital.

## Case report

The patient first presented to the emergency department of University Hospital Split, Križine, after five days of home isolation and COVID-19 treatment. He had a fever of up to 38.5 °C, myalgia, and diarrhea. As a medical doctor in the same hospital, the patient had been in contact with COVID-19 patients and coworkers. Medical history was unremarkable and no chronic medical conditions were reported. Before admission, the patient was treated for five days with antipyretics, which resolved fever, and azithromycin. Upon the first hospital presentation, he was eupneic, with blood oxygen saturation (Sao_2_) of 97%. The laboratory results indicated a barely increased C-reactive protein (CRP) of 11.3 mg/L. A chest x-ray showed bilateral basal and perihilar opacifications. The patient was released for home treatment, with prescribed 32 mg methylprednisolone for seven days in a de-escalating pattern.

**Figure 1:**
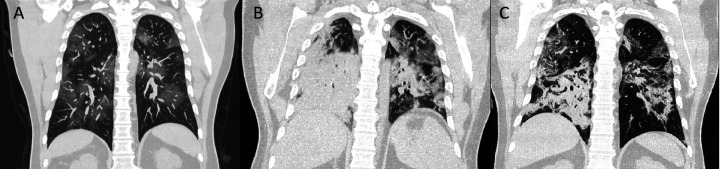
Chest computed tomography (CT) images (coronal reconstruction) at three time points. (**A**) Diffuse bilateral areas of ground glass opacities in all lobes at the first day of hospitalization. (**B**) Mixed airspace and ground glass opacities sparing only the lung apices at the thirteenth day of hospitalization. (**C**) Regression of airspace and ground glass opacities with fibrous changes in the right upper and middle lobe and left upper lobe at the twenty-third day of hospitalization.

Four days later, the patient visited the emergency department again, with worsening symptoms (fever over 39 °C and dry cough). In between the hospital visits, methylprednisolone dose was escalated to 64 mg and amoxicillin and clavulanic acid were added after finishing azithromycin. Sao_2_ upon the second presentation was 94%. Laboratory tests (Table 2) indicated elevated leukocytes (10.9 × 10^9^/L) with neutrophil predominance (90.4%, lymphocytes 5.7%) and mildly elevated CRP of 24.9 mg/L. A native chest CT showed extensive bilateral ground-glass opacities (Figure 1). Due to clinical and radiologic worsening, the patient was admitted to a specialized COVID-19 ward, where he was administered remdesivir (200 mg iv for five days), methylprednisolone 240 mg, enoxaparin 0.6 mL, moxifloxacin 400 mg, and supportive therapy. Oxygen supplementation was started the next day with 2 L/min nasal cannula. On the third day of hospitalization, blood oxygen saturation decreased (Sao_2_ 91%), and high-flow oxygen therapy (35 L/min) was started.

**Table 2:** The patient's key laboratory findings

Day of hospitalization	Leukocytes (10^9^/L)	PT	D-dimers (mg/L)	Albumin (g/L)	CRP (mg/L)	Procalcitonin (ng/mL)	Ferritin (ng/mL)	LDH (U/L)
1st day of the disease	4.50	/	0.39	/	11.3	/	/	227.0
1st (4th day of the disease)	10.90	1.23	0.65	39.9	24.9	/	/	445
6th	17.30	1.01	21.9	27.0	17.0	0.08	1535	663
7th	14.40	0.78	>35.2	24.4	17.0	0.07	/	/
8th	15.30	0.94	>35.2	25.7	62.0	/	/	475
8th	15.90	/	/	/	/	/	/	/
9th	14.20	0.92	>35.2	24.9	35.8	0.08	/	/
	First round of MSC							
10th	16.7	0.95	>35.2	/	23.6	0.03	850	/
11th	17.2	1	31.98	24.4	37.6	0.16	/	324
12th	16	1.08	13.42	24.9	23.7	0.11	1.285	292
	Second round of MSC							
12th	15.1	1.1	16.44	27.3	20.5	0.11	1.008	305
13th	14.8	1.09	/	30.4	17.9	0.11	/	269
14th	12.9	1.21	/	31.8	58.9	0.14	/	/
16th	12.5	0.97	/	/	31	0.08	/	/
	Third round of MSC							
17th	11.1	0.75	/	29.3	25.1	0.1	/	/
18th	12	1.03	/	31.1	28.2	0.1	/	/
19th	17.2	1.01	7.33	34.3	25.7	0.09	2.154	389
20th	12.5	0.94	/	31.5	127.0	0.18	/	/
21st	13.5	0.97	/	33.8	89.9	0.15	/	/
22nd	9.8	0.87	/	33.8	77.2	0.15	/	/
23rd	7.9	/	12.5	/	56.0	0.12	/	/
24th	7.5	1.06	/	/	40.1	0.15	/	/
25th	6.4	0.94	/	32.3	26.2	0.11	/	/
26th	7.4	0.92	/	36.9	24.6	0.08	/	260
28th	9.2	/	/	/	8.7	0.07	/	/
30th	9.6	0.95	5.26	43.1	4.3	0.09	837	235

*Abbreviations: PT – prothrombin time; CRP – C reactive protein; LDH – lactate dehydrogenase; MSC – mesenchymal stem cells; black line – period of mechanical ventilation.

On the sixth day of hospitalization, the patient was dyspnoic. Laboratory findings suggested an increased inflammatory response, with Sao_2_ 87% (Table 2). CT pulmonary angiography showed no signs of pulmonary embolism, but it did show a progression of bilateral consolidations (Figure 1). High-flow oxygen was increased to 45 L/min. On the same day, the patient was examined for severe thrombocytopenia (10 × 10^9^/L) by a hematologist, who diagnosed immunogenic thrombocytopenia, administered intravenous immunoglobulins (400 mg/kg for 5 days), and changed the dose of methylprednisolone to 100 mg.

On the seventh day of hospitalization, the patient was referred to the COVID-19 intensive care unit (ICU), where he was sedated, intubated, and started mechanical ventilation (synchronized intermittent mandatory ventilation f/24min, TV 440 mL; first FiO_2_ 100%, then gradually decreased to 80%) in a prone position, which improved blood oxygenation. On the same day, FiO_2_ was gradually reduced to 50%. Bronchoscopy, performed the following day, was unremarkable. The D-dimer levels were immeasurably high (>35.2 mg/L) (Table 2). The patient was given a dose of COVID-19 convalescent plasma (CCP). The plasma was obtained in the Croatian Institute of Transfusion Medicine from a healthy donor with a documented history of SARS-CoV-2 infection who had been asymptomatic for ≥28 days and was specified eligible according to standard blood donor criteria. SARS-CoV-2 neutralizing antibodies in CCP were quantified by infective-virus neutralization assay, using a reference calibrated according to the First WHO International Standard for anti-SARS-CoV-2 immunoglobulin (human) (NIBSC code 20/136, NIBSC, Potters Bar, UK), so the titer is expressed in international units per milliliter (IU/mL) (4). The volume of the CCP dose used for therapy was 200 mL, and the titer of neutralizing antibodies was 242 IU/mL.

Since all conventional and recognized therapies had been exhausted, the hospital ethics committee and drug committee approved the use of MSC compassionate treatment upon the request of the attending physicians and after obtaining written consent from the patient’s wife. During the ICU stay, the patient was given three doses of ImmunoART^TM^ (Educell Ltd, Trzin, Slovenia, member of Medical Biobank Swiss Institute SA), allogenic, HLA-incompatible, and non-related bone marrow-derived MSC. The first dose (10^6^ cells/kg) was administered intravenously on the ninth day of hospitalization. MSC doses were prepared from the bone marrow of a young, healthy donor, who agreed to make the donation for allogeneic treatment. The donor was negative for viral markers (HBs Ag, HBc Ab, HCV Ab, HIV 1-2 Ab, TPHA, HBV NAT, HCV NAT, HIV NAT) according to EU legislation, Directive 2004/23/EC of the European Parliament and the Council of the European Union (the body setting the quality and safety standards for donation, procurement, testing, processing, preservation, storage, and distribution of human tissues and cells). The cells were prepared for hospital exemption use in a controlled and verified laboratory in a cleanroom facility in a class A safety cabinet following the guidelines and principles of good manufactured practice. The cultivated cells used in therapy showed morphological characteristics of MSC and expressed CD105, CD73, and CD90, but not CD45 and CD34. The patient's laboratory parameters were monitored daily, showing a decrease in D-dimer and CRP levels in a three-day period (Table 2).

The second MSC dose was given on the twelfth day of hospitalization. The treatment was continued in the non-COVID ICU, University Hospital Split, Firule, since the patient was no longer considered contagious. This is a standard protocol that allows freeing up hospital beds for the acutely ill contagious patients. The therapy and mechanical ventilation were maintained.

The next day, despite a marked reduction in leukocyte count and CRP level (Table 2), Sao_2_ fell below 90%. Bronchoscopy revealed an abundant yellowish secretion, which was sent for microbiologic evaluation. A repeated native CT of the chest showed extensive airspace opacifications and pneumomediastinum (Figure 1). Ventilation compliance was low (tidal volumes below 350 mL), with decreasing Sao_2_ levels, which is why ARDS was considered the leading diagnosis. A recruitment maneuver was performed, and ventilation with high positive end-expiratory pressure (PEEP) was initiated (Intellivent ASV, Hamilton, Bonaduz, Switzerland) with low driving pressure (below 14 cm H_2_O). The same night, a subcutaneous emphysema was observed. PEEP was continuously reduced in the following days, with careful monitoring of the respiratory parameters. The microbiologic culture of the bronchial aspirate taken on the twelfth day was positive for methicillin-resistant *Staphylococcus aureus* and *Acinetobacter Baumannii*. Antibiotic treatment was started immediately based on the antibiogram (colistin 9 million i.u. initially, the next and following days 2 × 4.5 million i.u., fosfomycin 3 × 8 g, and linezolid 2 × 600 mg). Repeated bronchoscopy on the fifteenth day showed the same findings, and no reduction in subcutaneous emphysema was visible on the chest x-ray. The third MSC dose was given on the sixteenth day of hospitalization. A chest x-ray showed the regression of opacifications. Later the same day, the patient experienced paroxysmal hypertension >200 mm Hg and tachycardia >140/min. Echocardiography, although impeded by pneumomediastinum, showed normal left ventricle morphology with an ejection fraction of 50%. On the seventeenth day, the patient was extubated and put on high-flow oxygen (60 L/min), with satisfying peripheral blood oxygen saturation.

The clinical condition continued to improve, and oxygen supplementation needs were lowering, with only nasal cannula being sufficient on the nineteenth day of hospitalization. The patient was also able to verticalize in the course of physical therapy.

On the twentieth day, the patient complained of lower urinary tract pain. Urine samples (bacteriuria) suggested a urinary tract infection. Since blood cultures were positive for gram-negative bacilli, ceftazidime/avibactam (3 × 2.5 g iv) therapy was started.

In the following days, the condition was stable. Leukocytes returned to physiological levels on the twenty-second day of hospitalization. A chest CT performed on the twenty-third day of hospitalization showed a regressive dynamic of airspace opacifications (Figure 1). CRP levels continued to decline and were normal on the thirtieth day of hospitalization, when all antibiotics were discontinued. The patient was able to perform basic physical activities on his own, without the need for oxygen supplementation, and on the thirty-first day of hospitalization was transferred to a physical medicine clinic. The patient remained in the hospital for an additional observation and rehabilitation period, after which he was discharged on the forty-ninth day of hospitalization.

## Discussion

This case study describes the clinical course of the first Croatian patient treated with MSC for severe COVID-19. The patient's clinical condition progressively worsened since admission and he eventually required mechanical ventilation. The treatment consisted of corticosteroids, immunoglobulins, and convalescent plasma, all of which were indicated due to their anti-inflammatory properties. No Croatian national guidelines on the use of CCP are currently available. In the present case, CCP was used due to its possible immunomodulatory effects, rather than due to its neutralizing antiviral effects (5). This decision was based upon FDA recommendations that the possible benefits of CCP outweigh the risks in critically ill COVID-19 patients (6). Remdesivir was also used, for 5 days.

Since the conventional therapeutic approach was unsuccessful, compassionate use of MSC therapy was initiated, following the Article 83 Regulation EC No 726/2004 of the European Parliament and Council of the European Union. Upon request of the attending physicians in University Hospital Split, Križine, and the patient’s family, MSC were obtained from a young, living donor, and prepared as described previously. After our initial contact with Educell Ltd, all the relevant documentation on ImmunoART^TM^ MSC harvesting, expansion, and delivery methods, as well as the opinion of the Slovenian drug regulating agency, was provided to the ethics committee of University Hospital Split. Croatian Ministry of Health was informed about the planned procedure. Meanwhile, Educell Ltd declared the use of MSC for humanitarian purposes, thereby providing them free of charge. No additional approvals were requested after the transfer to University Hospital Split, Firule, as both hospitals are considered a single administrative unit.

MSC are pluripotent stem cells differentiating into different mesodermic and non-mesodermic lineages. Their secretome consists of immunomodulatory cytokines and chemokines, which were previously shown to modulate endothelial and epithelial permeability, promote endothelial repair, decrease scarring, and act anti-inflammatory (7). Autologous MSC are used in orthopedics for the treatment of osteoarthritis and have shown good results in pain reduction and increasing joint mobility, without any observed treatment-specific side effects (8-10).

MSC are immune-privileged cells with a strong immunomodulatory (via IL-10, TGFb, IDO, PGE2), antimicrobial (via antimicrobial peptide LL-37), and regenerative properties (via VEGF, KGF, EGF) (11,12). Preclinical studies have shown that after iv administration MSC remain “trapped” in the lung during the first passage, so, in the context of ARDS and COVID-19, they accumulate in the target tissue (13). Besides, MSC modulate the immune response via macrophages transition from an inflammatory M1 into an anti-inflammatory M2 phenotype (14,15). In addition, animal models showed the potential of their use for virus-induced pneumonia, ARDS, and lung failure treatment (16). At the moment of writing, *clinicaltrial.gov* lists 72 registered trials on MSC use in COVID-19 patients (17). Phase-one clinical trials have proven MSC therapy to be well tolerated and safe (11). A recent application of adipose tissue-derived MSC in 13 patients with severe COVID-19 pneumonia on medical ventilation significantly reduced the inflammatory parameters (CRP, IL-6, ferritin, LDH, D-dimer) (18). These findings are in line with those demonstrated in a systematic review and meta-analysis by Qu et al, who found no related serious adverse events after intravenous or intratracheal administration of allogenic MSC of various origin, as well as observed an improved lung function, radiographic findings, and inflammatory biomarkers level (19).

**Figure 2:**
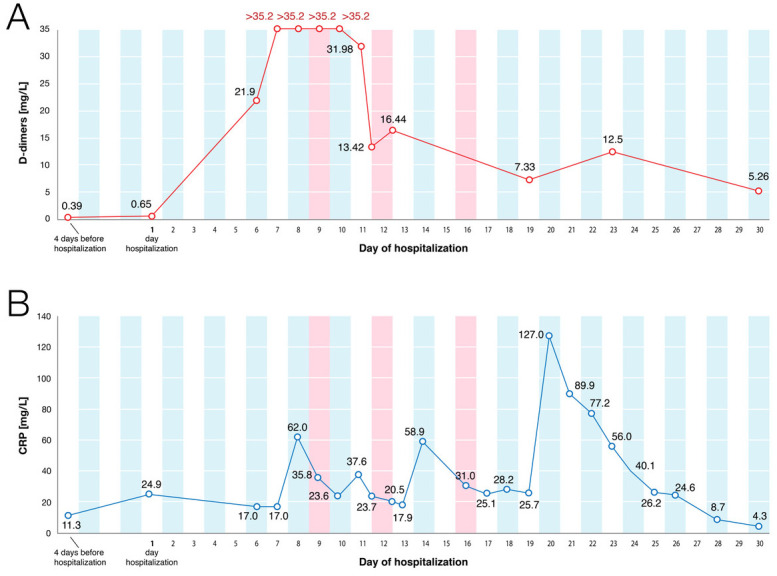
Inflammatory parameters by the day of hospitalization. (**A**) C-reactive protein (CRP); (**B**) D-dimers. The red lines represent the days when mesenchymal stem cells were applied.

In this case, after MSC administration we observed a reduction in leukocyte count, D-dimer levels, and CRP-levels, all of which are prognostic factors for COVID-19 severity (20-23) (Table 2, Figure 2). The D-dimer reduction was particularly impressive. A recent multicentric study demonstrated that elevated D-dimer levels (2.025 mg/L cutoff) correlate with poor prognosis (24). Despite the increased D-dimer levels, pulmonary angiography showed no signs of pulmonary embolism. However, the first MSC dose was applied a day after the convalescent plasma application, which can confound the interpretation of the observed effect. Nevertheless, the progressive clinical deterioration and the ARDS development were not stopped. The third MSC dose was applied on the sixteenth day of hospitalization and coincided with the radiographic regression of ARDS. The following day, the patient was extubated and switched to high-flow oxygen. These observations lead us to believe that MSC therapy may show a visible effect in a 24-hour period after administration, but the treatment is potentially only effective in the setting of acute inflammatory conditions driven by cytokine storm, such as ARDS. However, we cannot confirm this observation because the required biomarkers were unavailable for analysis. This case did, however, demonstrate that compassionate MSC application was well tolerated after three treatment rounds, as no adverse events were observed that could be directly attributed to the procedure. Similar results were demonstrated in a case series by Hashemian et al, who reported a positive clinical effect (improved Sao_2_ and dyspnea) in 5 out of 11 patients who received prenatal (umbilical cord- and placenta-derived) MSC infusion in a two- to three-day period (25). The authors also reported a decrease in proinflammatory parameters (TNF-alpha, IL-6, IL-8, CRP) on the fifth day of treatment (24 hours after the third dose of MSC). These parameters were not monitored in our patient, so we are unable to compare the results of the two studies (25). A prolonged hospital stay in COVID-19 patients can be complicated by hospital-acquired bacterial superinfections (26,27). The exact incidence of superinfections in Croatian hospitals has not been established, but they affect both the patient’s prognosis and length of hospital stay (28). Our patient had a superinfection with *S. aureus* and *A. baumanii* confirmed in the bronchial aspirate upon bronchoscopy on day 12. We effectively treated the patient with colistin, fosfomycin, and linezolid. The second bacterial infection was found in blood cultures (gram-negative bacilli) on day 20, after the patient was already extubated and clinically improving. Ceftazidime/avibactam therapy was successful, and the patient’s leukocyte count decreased to physiological levels in two days. These findings indicate the need for special attention when it comes to bacterial superinfections in COVID-19 patients, as they already have a weakened immune system. In our experience, the isolated specimens did not differ between COVID-19 and non-COVID-19 patients.

The main study limitations were the ability to obtain only the laboratory parameters used in daily clinical practice, which prevented us from analyzing the levels of cytokines driving the inflammatory response. Second, the use of convalescent plasma, remdesivir, and immunosuppressive drugs before MSC application prevent us from drawing independent conclusions on MSC efficacy. We were unable to exclude these factors, as MSC therapy has never before been used for COVID-19 treatment in Croatia, and the standard treatment protocols include the mentioned medications. Other limitations include a short follow-up and our inability to draw definite conclusions since the study was limited only to a single case. The observed effect of MSC in combination with readily used therapeutic measures should be further studied in larger patient cohorts and in centers able to perform more sophisticated laboratory analyses. However, since there is no currently available treatment for severe COVID-19, we propose MSC to be considered in patients who do not respond to commonly used therapeutic options.
